# Bioinformatics analysis of vascular RNA-seq data revealed hub genes and pathways in a novel Tibetan minipig atherosclerosis model induced by a high fat/cholesterol diet

**DOI:** 10.1186/s12944-020-01222-w

**Published:** 2020-03-25

**Authors:** Yongming Pan, Chen Yu, Junjie Huang, Yili Rong, Jiaojiao Chen, Minli Chen

**Affiliations:** Comparative Medical Research Institute, Experimental Animal Research Center, Academy of Chinese Medical Sciences, Zhejiang Chinese Medical University, No. 548 Binwen Road, Binjiang District, Hangzhou, 310053 China

**Keywords:** Hypercholesterolemia, Atherosclerosis, Tibetan minipig, Transcriptome, Inflammatory

## Abstract

**Background:**

Atherosclerosis is a major contributor to cardiovascular events, however, its molecular mechanism remains poorly known. Animal models of atherosclerosis can be a valuable tool to provide insights into the etiology, pathophysiology, and complications of atherosclerosis. In particular, Tibetan minipigs are a feasible model for studying diet-related metabolic and atherosclerotic diseases.

**Methods:**

We used vascular transcriptomics to identify differentially expressed genes (DEGs) in high fat/cholesterol (HFC) diet-fed Tibetan minipig atherosclerosis models, analyzed the DEGs gene ontology (GO) terms, pathways and protein-protein interactions (PPI) networks, and identified hub genes and key modules using molecular complex detection (MCODE), Centiscape and CytoHubba plugin. The identified genes were validated using the human carotid atherosclerosis database (GSEA 43292) and RT-PCR methods.

**Results:**

Our results showed that minipigs displayed obvious dyslipidemia, oxidative stress, inflammatory response, atherosclerotic plaques, as well as increased low-density lipoprotein (LDL) and leukocyte recruitment after 24 weeks of HFC diet feeding compared to those under a regular diet. Our RNA-seq results revealed 1716 DEGs in the atherosclerotic/NC group, of which 1468 genes were up-regulated and 248 genes were down-regulated. Functional enrichment analysis of DEGs showed that the HFC diet-induced changes are related to vascular immune-inflammatory responses, lipid metabolism and muscle contraction, indicating that hypercholesterolemia caused by HFC diet can activate innate and adaptive immune responses to drive atherosclerosis development. Furthermore, we identified four modules from the major PPI network, which are implicated in cell chemotaxis, myeloid leukocyte activation, cytokine production, and lymphocyte activation. Fifteen hub genes were discovered, including TNF, PTPRC, ITGB2, ITGAM, VCAM1, CXCR4, TYROBP, TLR4, LCP2, C5AR1, CD86, MMP9, PTPN6, C3, and CXCL10, as well as two transcription factors (TF), i.e. NF-ĸB1 and SPI1. These results are consistent with the expression patterns in human carotid plaque and were validated by RT-PCR.

**Conclusions:**

The identified DEGs and their enriched pathways provide references for the development and progression mechanism of Tibetan minipig atherosclerosis model induced by the HFC diet.

## Introduction

Hypercholesterolemia and atherosclerosis are major lifestyle-related metabolic disorders and health problems in modern society [1]. Their occurrences are known to be related to genetic and environmental factors [2, 3]. In specific, hypercholesterolemia is an important risk factor for atherosclerosis, which can lead to atherosclerosis by inducing a local inflammatory response, oxidative stress damage, and damages to vascular endothelial cell metabolism and function. Recently, studies have suggested that imbalances in nutrient intake may lead to hypercholesterolemia and atherosclerosis [4, 5]. However, a lack of appropriate animal models has made it difficult to assess such an association between hypercholesterolemia and atherosclerosis caused by dietary patterns. Rodents are not an appropriate model for atherosclerosis in humans, because they have such a different lipoprotein profile that atherosclerosis rarely occurs in rodents unless they are genetically modified or fed to an extreme diet [5]. Moreover, in these models, genetically induced hypercholesterolemia usually leads to cholesterol levels that are much higher than those in humans. In particular, mouse models have been challenged recently for studying human inflammatory/immune responses [6]. Therefore, the establishment of an ideal experimental animal disease model is essential for studying the pathophysiology of atherosclerosis.

Pigs, which can spontaneously form atherosclerosis, provide a more representative animal model for studying dyslipidemia and atherosclerosis [7, 8]. The physiological and anatomical aspects of pigs in lipid metabolism, cardiovascular metabolism and digestion are very similar to those in humans. Furthermore, the omnivorous nature of pigs makes them highly suitable for studying dietary-based patterns and for translating findings from pigs to humans [5]. In particular, Tibetan minipigs have many advantages as a model for studying diet-related cardiovascular metabolic diseases [9, 10]. Our previous studies have shown that Tibetan minipigs are a viable model for studying atherosclerosis induced by dietary patterns. They can form atherosclerosis with 24 weeks of a high-fat/cholesterol diet, and have characteristics like lipid disorders, insulin resistance, hypertension and cardiac dysfunction [9]. The lipid metabolism changes and atherosclerotic lesions in the Tibetan minipig model are also very close to those in humans, but the underlying molecular pathological mechanisms are not yet clear.

Recently, it has been recognized that the relationship between dietary patterns and cardiovascular diseases is complex, which involves many changes in biological systems [11]. The increase in genomic information, especially after the emergence of high-throughput sequencing technologies, provides an opportunity to better understand the intricate biological processes of the disease. Previous studies have revealed molecular changes in rabbit and human atherosclerosis at the mRNA level [12, 13]. Recently, hypercholesterolemia has been found to stimulate immune responses and may promote inflammatory diseases [14, 15]. Herein, we utilized Tibetan minipigs fed with a high-fat/cholesterol diet to mimic human hypercholesterolemia and atherosclerosis associated with today’s poor dietary patterns. We used vascular transcriptome to identify the extent of changes in vascular gene expression, and performed bioinformatics analysis to discover potential hub genes and key pathways caused by atherosclerosis. This study therefore provided clues about the molecular mechanism of atherosclerosis, revealed new therapeutic targets and biomarkers, and can serve as a reference for future research and application of Tibetan minipigs in studying human atherosclerotic diseases.

## Materials and methods

### Animals and experimental protocol

Before the study, the experimental protocol was approved by the Animal Care and Use Committee at the Zhejiang Chinese Medical University (approval number: ZSLL-2016-0031). Ten male Tibetan minipigs (3–4 months old) were purchased from Guangdong Pearl Lab Animal Sci & Tech Co., Ltd. All minipigs were kept in the animal house of Experimental Animal Research Center of Zhejiang Chinese Medical University at 20–22 °C and with a 12 h light/dark cycle. After 4 weeks of environmental accommodation, the animals were divided into a normal control (NC) group and an atherosclerosis model group with 5 animals per group. The atherosclerosis model was established by feeding minipigs with a high-fat/cholesterol (HFC) diet (containing 15% shortening oil, 1.5% cholesterol, 10% egg yolk powder, and 73.5% basal feed) for 24 weeks, as we previously described [9]. Minipigs were euthanized at the 24th week.

### Changes in biochemical parameters and white blood cell (WBC) count

Following a 16 h fast at the 24th week of a HFC diet, the body weights of minipigs were measured. Heparin anticoagulant blood was collected from the anterior vena cava of minipigs, and was then centrifuged for plasma collection. Total cholesterol (TC), triglycerides (TG), high-density lipoprotein-cholesterol (HDL-C), and low-density lipoprotein-cholesterol (LDL-C) were measured (Hitachi 7020 automatic biochemical analyzer) as previously described [9]. Atherogenic index (AI) was derived from the ratio of (TC–HDL-C) to HDL-C. Plasma C-reactive protein (CRP) and tumor necrosis factor-α (TNF-α) levels were detected using ELISA kits (Nanjing Jiancheng Bioengineering Institute, China). In addition, 1 mL of EDTA-K_2_ anticoagulant blood was taken, and the components of the blood cells including the WBC count were analyzed on an automated hematology analyzer (2120, Bayer Healthcare Ltd., USA).

### Atherosclerosis lesion analysis

At the end of the study, all minipigs were sacrificed for autopsy. The blood vessels from the aortic arch to the abdomen of the descending aorta (flat 3–4 lumbar vertebrae) were fixed with 10% buffered formalin, stained with 0.5% Sudan IV, and then photographed. Image J software was used to quantify Sudan IV-stained area, expressed as a percentage of the total surface area. The arterial vessels at the end of the abdominal aorta were placed into 10% paraformaldehyde solution, embedded in paraffin, cut into 5-μm-thick sections, stained with hematoxylin-eosin (H&E) and Masson’s trichrome, and immunohistochemically stained with NF-ĸB p65 subunit, VCAM1, and MMP9 antibodies (1:300 dilution, NF-ĸB p65,SC-8008; VCAM1, SC-18854; MMP9, SC-21733; Santa Cruz Biotechnology, CA, USA). Image pro plus 6.0 software was used to measure the intima-media thickness (IMT) and the staining percentage of the positive area [9]. In addition, immunofluorescent staining was performed with smooth muscle actin antibody (α-SMA) and CD68 antibody (1:200; α-SMA, SC-32251; CD68, SC-70761; Santa Cruz Biotechnology, CA, USA), which was then secondarily labeled with Alexa fluor 647-conjugated goat anti-rabbit IgG (ab150087) and Alexa fluor 488-conjugated goat anti-mouse IgG (ab150117) (Abcam, Cambridge, UK) respectively, and mounted with Fluorescent with DAPI. Slides were examined with a fluorescence microscope (VS120, OLYMPUS, Japan).

### Construction and sequencing of messenger RNA (mRNA) library

We randomly picked three pigs in every group for deep sequence analysis. Trizol reagent was used for RNA isolation from blood vessels at the end of abdominal aorta (Invitrogen, CA, USA). RNA quantity and purity were assessed using a Bioanalyzer 2100 and RNA 6000 Nano Lab Chip Kit (Agilent, CA, USA) with RNA integrity number (RIN) > 7.0. Ten micrograms of total RNA was used to isolate Poly (A) mRNA with poly-T oligo attached magnetic beads (Invitrogen). After purification, the mRNA was cleaved into small fragments by divalent cation at high temperatures. According to the protocol of the mRNA Seq sample preparation kit (Illunina, San Diego, USA), the RNA fragments after cleavage were reverse-transcribed to generate the final cDNA library. The average insertion size of the paired-end library was 300 bps. We then performed the paired-end sequencing on Illumina Hiseq 4000 (LC Sciences, Hangzhou, China) as recommended by the supplier. RNA-Seq data have been submitted to the Gene Expression Omnibus (GEO) database (Accession no.GSE140412).

### Transcriptome data analysis

Clean reads were obtained and mapped to the pig reference genome (Scrofa 11.1) using the HISAT package (version 2.0) with default parameters. Each sample’s mapped reads were assembled using StringTie (version 1.3.0). All the transcriptomes in the sample were merged to reconstruct a comprehensive transcriptome using perl scripts. The average FPKM (Reads per kilobase of exon per million) were calculated using StringTie. Differentially expressed genes (DEG) were selected with log_2_ (fold change) > 1 or log_2_ (fold change) < − 1 and with statistical significance (*p-*value < 0.05) using edgeR.

### Gene ontology (GO) and Kyoto encyclopedia of genes and genomes (KEGG) enrichment analysis of DEGs

To assess the functions of the identified DEGs, g: profiler (https://biit.cs.ut.ee/gprofiler) was used to identify the enriched GO biological processes [16]. The results were integrated using the Enrichment Map [17], in which *p*-value < 0.01 was adjusted to indicate that the difference is statistically significant. KEGG Orthology Based Annotation System (KOBAS 3.0) (http://kobas.cbi.pku.edu.cn) was used to identify the enriched KEGG pathway based on adjusted *P* values, in which *p*-value < 0.05 was adjusted to indicate that the difference is statistically significant. In addition, Gene Set Enrichment Analysis (GSEA, version 4.0.3, the broad institute of MIT and Harvard) was used to assess whether a genetically defined genome is statistically significant between the two phenotypes [18]. GSEA was performed to identify enriched hallmark gene sets pathways between atherosclerosis and NC (FDR < 0.05), where the hallmark gene sets were collected from the Molecular Signature Database (MSigDB).

### Identification of modules and hub genes in PPI networks

DEGs were uploaded to the STRING database to build a PPI network [19], with a confidence score ≥ 0.4 as the cut-off criterion. The PPI network was then visualized in Cytoscape software 3.7.2 [20]. The PPI network centrality parameters (degree, betweenness, eigenvector) was analyzed using CentiScaPe 2.2 similar to previous studies [21, 22]. In this work, the average values of degree, betweenness, and eigenvector were calculated. To identify nodes with higher centrality values for the three selected parameters, a Venn diagram was drawn using Venny 2.1 software (https://bioinfogp.cnb.csic.es/tools/venny/). Finally, the major PPI network was constructed based on the intersection of three centrality parameters in the Venn diagram. The most significant modules were identified from the major PPI network using the Molecular Complex Detection (MCODE) plugin. K-score = 5 was set as the criterion for MCODE analysis. In addition, we used the CytoHubba plugin and the degree topological analysis [23] to identify hub genes, where genes of the top 15 degrees were considered to be hub genes in the network. Transcription factor prediction was performed using the iRegulon plugin of Cytoscape 3.7.2 [24].

### Hub genes validation

We validated the RNA-seq results of 9 genes by real-time PCR. Briefly, complementary cDNA was synthesized using a PrimeScript™ RT reagent kit (Takara, Dalian, China). Table 1 shows the sequences of the used primers. Real-time PCR was performed on Step one plus using SYBR Premix Ex Taq Reagent Kit (Takara, Dalian, China). GAPDH was used as a house-keeping reference to calculate the relative expression of target genes with the 2^-ΔΔCt^ method. To confirm the expression patterns of the15 hub genes in human atherosclerosis, we used the GEO database of human carotid atherosclerotic plaques (GSE43292, containing 32 atheroma plaque samples and 32 adjacent carotid tissue) for validation.

**Table 1:** Primers for quantitative real-time PCR analysis

Gene	Forward	Reverse	Product size	Accession no.
*TNF*	CCACGCTCTTCTGCCTACTGC	CTCGGCTTTGACATTGGCTAC	161	NM_214022.1
*ITGB2*	GTCAGGCGGCCACGTTCAAC	TGAGGTCGTCGAGCATGGAGTAG	102	NM_213908.1
*VCAM1*	CAGATCCACGCTGGTCATGAATCC	GACACCTGACTGTAACTGGCTTCC	185	NM_213891.1
*TYROBP*	ATCTGGTGCTGACCCTCCTC	CCTCTGTGTGTTGAGGTCGC	176	NM_214202.1
*LCP2*	CCGAACAGAGGCAGAAGCTG	TTCCCTCGGAGTCCAGTTCC	192	XM_021077083.1
*SPI1*	CTTGTCCACCCACCAGATGC	GGGAGACAGGCAGCAAGAAG	98	NM_001001865.1
*CTSS*	GGTGCTCCTCCTGTGCTCCTC	GATGAGACGCCGTGCTACTTCC	136	XM_021089893.1
*PTPN6*	CCTCCCGCACCTCCTCCAAG	GTCTGCTGACCGCTGCTTCTTC	98	EU753189.1
*GAPDH*	CCATCACCATCTTCCAGGAGCGAG	AAGTTGTCATGGATGACCTTGGCCA	286	NM_001206359.1

### Statistical analysis

All data are expressed as means ± SEM. Statistical analysis was performed with GraphPad Prism 7.0 software (GraphPad Software Inc., USA). The Mann-Whitney test was used to compare between the two groups. *P* < 0.05 indicates statistical significance.

## Results

### Changes of clinical phenotypic characteristics and histopathological assessment in Tibetan minipigs with a HFC diet

**Fig. 1:**
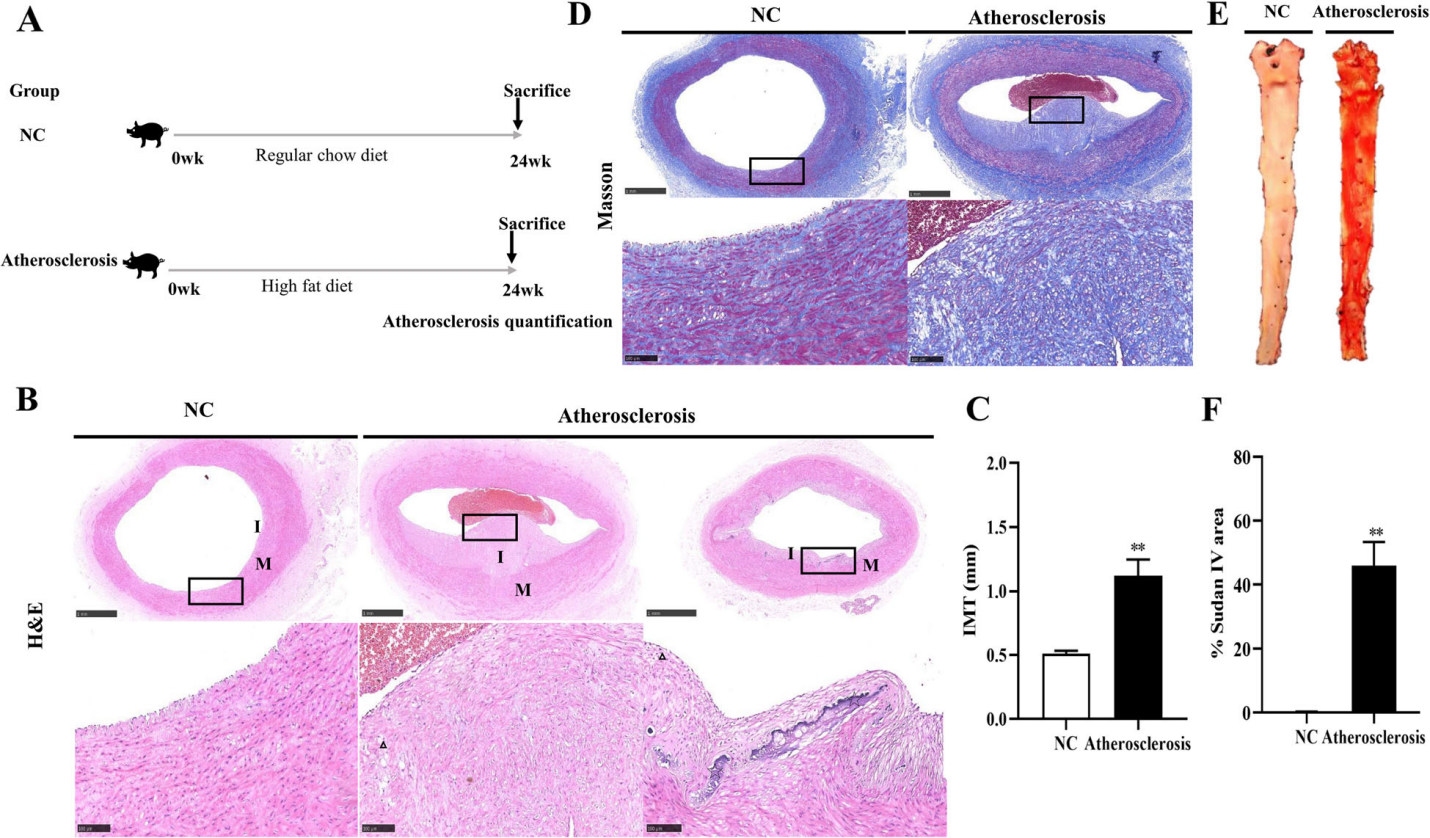
Artery vascular pathological assessment in Tibetan minipigs after 24 weeks of continuous feeding with a HFC diet. **a** Study protocol. 10 Tibetan minipigs were randomly allocated to NC and atherosclerosis groups and fed with a regular diet or a HFC diet throughout the study, and the minipigs were sacrificed at 24 weeks later. **b** H&E staining of the abdominal aorta and the images of the second line are an enlarged view of the red box in the first line. Triangle represents foam cells, pentagram indicates vascular calcification. **c** IMT of the abdominal aorta. **d** Masson staining of the abdominal aorta and the images of the second line are an enlarged view of the red box in the first line. **e** Aortic lipid analysis by Sudan IV staining. **f** Percentage of Sudan IV area. Data are expressed as means ± SEM (*n* = 5). *P < 0.05, **P < 0.01

We saw from the H&E staining that the abdominal aortic intima was thickened in the atherosclerosis group, with a large number of inflammatory cells infiltrating, calcifying and forming foam cells (Fig. 1b). In specific, our quantitative analysis revealed a significant increase of IMT increased in the atherosclerosis group (*P* < 0.01, Fig. 1c). Masson staining showed that the content of collagen fibers in the intima and the media grew significantly after 24 weeks of HFC diet induction (Fig. 1d). From Sudan IV staining, we saw a significant increase of aortic lipid deposition in the atherosclerosis group compared with the NC group (*P* < 0.01, Fig. 1e-f). Meanwhile, significant changes were also observed in clinical phenotypic characteristics, as shown in Table 2. The body weight, TC, LDL-C, HDL-C, AI, WBC counts, as well as CRP and TNF-α levels, were significantly increased in the atherosclerosis group compared to the NC group (*P* < 0.05, *P* < 0.01), except for TG.

**Table 2:** Clinical biochemical indicators in Tibetan minipigs at 24 weeks of high-fat/cholesterol diet induction

Parameters	NC	Atherosclerosis
Body weight (kg)	27.60 ± 2.00	39.80 ± 0.84**
TC (mmol/L)	1.51 ± 0.08	13.06 ± 2.7**
TG (mmol/L)	0.31 ± 0.04	0.25 ± 0.03
HDL-C (mmol/L)	0.55 ± 0.03	2.89 ± 0.25**
LDL-C (mmol/L)	0.65 ± 0.03	8.35 ± 2.02**
AI	1.75 ± 0.11	3.38 ± 0.55**
WBC (10^9^/L)	11.46 ± 1.02	15.72 ± 1.53*
CRP (mg/L)	13.75 ± 1.54	24.66 ± 2.98*
TNF-α (ng/L)	146.70 ± 17.32	239.6 ± 26.84*

Data are expressed as means ± SEM (*n* = 5)

*P < 0.05, **P < 0.01 versus NC group

### Identification of differential genes and analysis of their functions and pathways

**Fig. 2:**
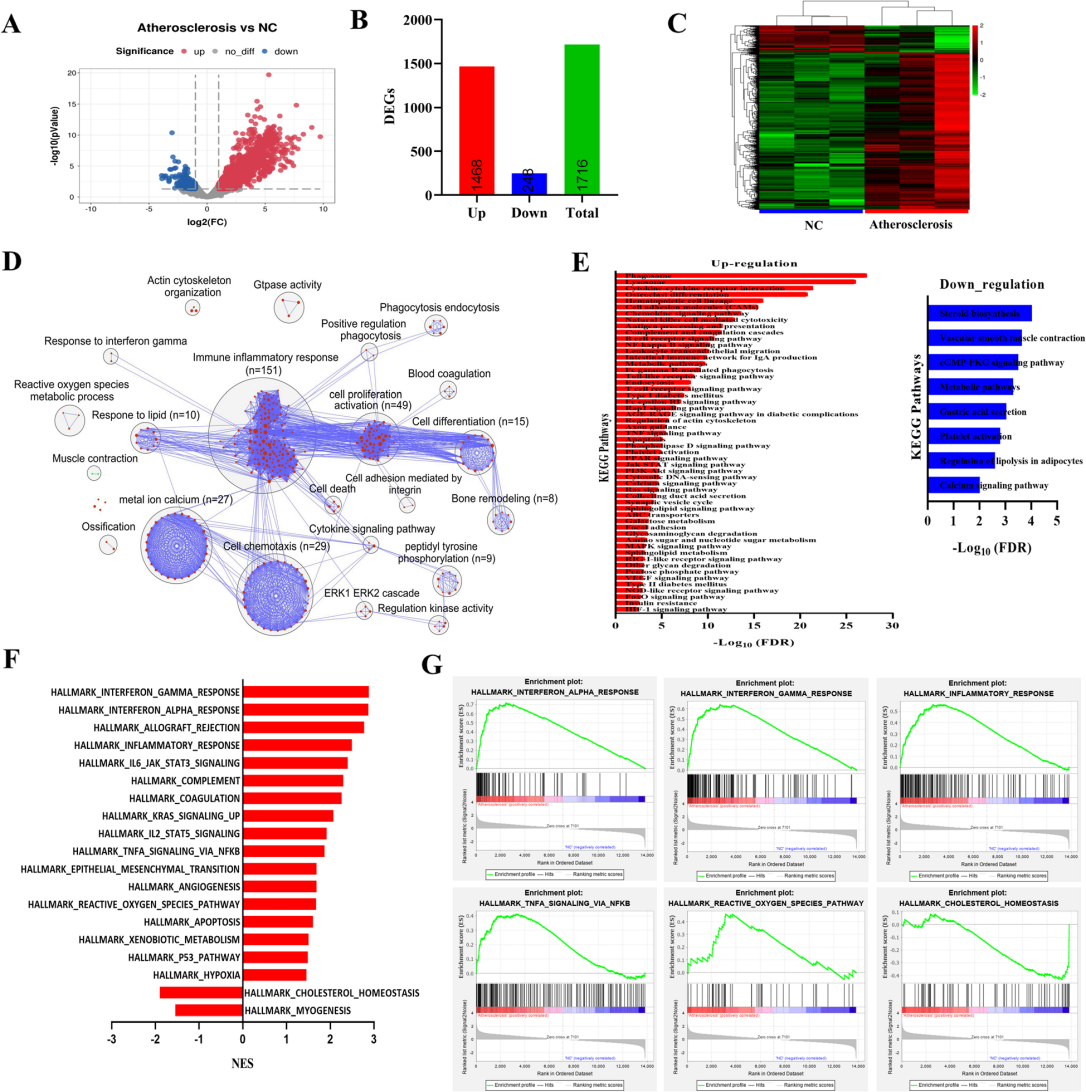
Identifying DEGs by transcriptome sequencings and functional analysis. **a** The volcano plots of DEGs in the atherosclerosis/NC gene set. The red dots represented up-regulated genes, and the blue ones represented down-regulated genes, and the gray ones showed no significance. **b** The numbers of DEGs up-regulated and down-regulated in the atherosclerosis/NC group. **c** Two-way hierarchical clustering heatmap of up-regulated and down-regulated DEGs in the atherosclerosis/NC group. **d** The enrichment maps of GO biological processes (FDR q value < 0.01, *p*-value< 0.005) display the enriched genes-sets between atherosclerosis and NC by g: profiler online tool, the red nodes indicates up-regulated DEGs, the green indicates down-regulated DEGs. **e** The KEGG pathway analysis (FDR q value < 0.01) of up-regulated and down-regulated DEGs in the atherosclerosis/NC group by KOBAS 3.0 online analysis database. **f** Hallmark gene sets enriched in the atherosclerosis/NC group by GSEA (FDR q value< 0.05). **g** GSEA enrichment plots for six hallmark gene sets enriched in the atherosclerosis/NC group

To observe the changes of vascular transcriptome in the Tibetan minipig atherosclerosis model, six cDNA libraries were constructed from high-throughput sequencing of three abdominal aortic samples from each group (*N* = 3). One thousand seven hundred sixteen DEGs were identified in the atherosclerosis/NC group, including 1468 up-regulated genes and 248 down-regulated genes (Fig. 2a-c).

To understand the biological function of atherosclerosis in Tibetan minipigs induced by the HFC diet, the g: profiler online tool was used to identify overrepresented biological pathways, and the Enrichment Map plugin was used for enrichment visualization (Fig. 2d). These results showed that the up-regulated DEGs are markedly enriched in many biological processes including immune-inflammatory response, cell proliferation activation, cell chemotaxis, metal ion calcium, cell differentiation, response to lipid, bone remodeling, reactive oxygen species metabolic process, and cell death, whereas the down-regulated DEGs are enriched in muscle contraction. In KEGG analysis (shown in Fig. 2e), we saw that the up-regulated DEGs are enriched in cytokine-cytokine receptor interaction, phagosome, lysosome, hematopoietic cell lineage, cell adhesion molecules (CAMs), natural killer cell mediated cytotoxicity, antigen processing and presentation, chemokine signaling pathway, complement and coagulation cascades, B cell receptor signaling pathway, NF-kappa B signaling pathway, T cell receptor signaling pathway, Fc gamma R-mediated phagocytosis, Toll-like receptor signaling pathway, TNF signaling pathway, PPAR signaling pathway, JAK-STAT signaling pathway and insulin resistance, while the down-regulated DEGs are enriched in steroid biosynthesis, vascular smooth muscle contraction, metabolic pathway, calcium signaling pathway, cGMP-PKG signaling pathway. In addition, gene set enrichment analysis (GSEA) method was used to identify the gene set hallmarks. The results showed that there were 17 pathways activated in the atherosclerosis/NC gene set, including interferon alpha response, interferon gamma response, inflammatory response, complement, coagulation, TNFα signaling via NFKB, reactive oxygen species pathway and apoptosis, while cholesterol homeostasis and myogenesis were significantly inhibited (Fig. 2f and g).

### Hub genes and key network identification in the PPI network

**Fig. 3:**
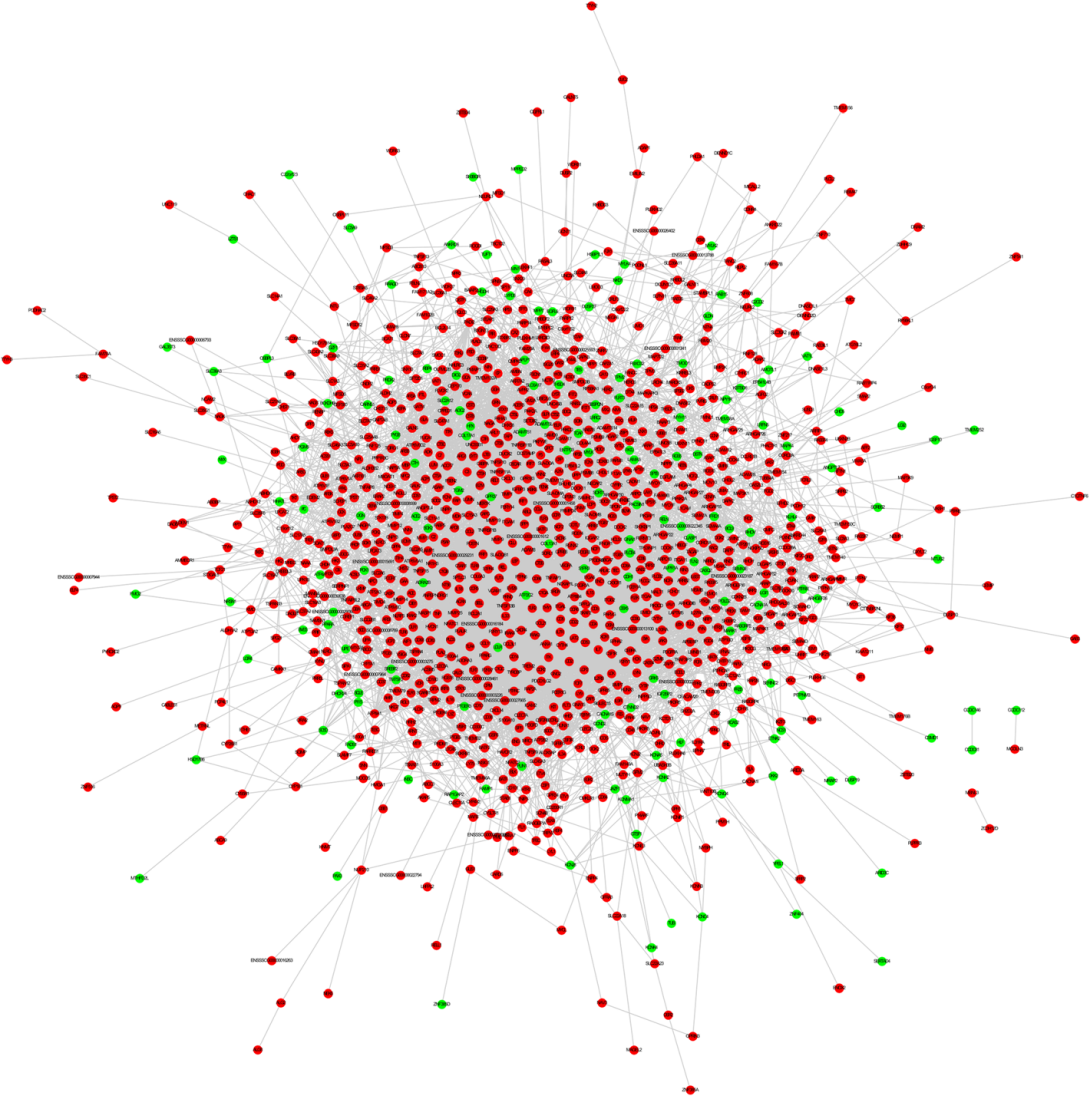
The initial PPI network of DEGs in the Tibetan minipigs atherosclerotic model induced by a high fat/cholesterol diet was constructed by the STRING database. Red nodes represent the up-regulated genes, while green nodes represent the down-regulated genes

To investigate the interaction of these 1716 DEGs in the atherosclerosis/NC gene set, an initial PPI network was created using STRING 11.0. The threshold of interaction score was set to > 0.4 (medium confidence), and the unconnected nodes were removed from the network. The initial PPI network consisted of 7137 edges and 1236 nodes (Fig. 3).

**Fig. 4:**
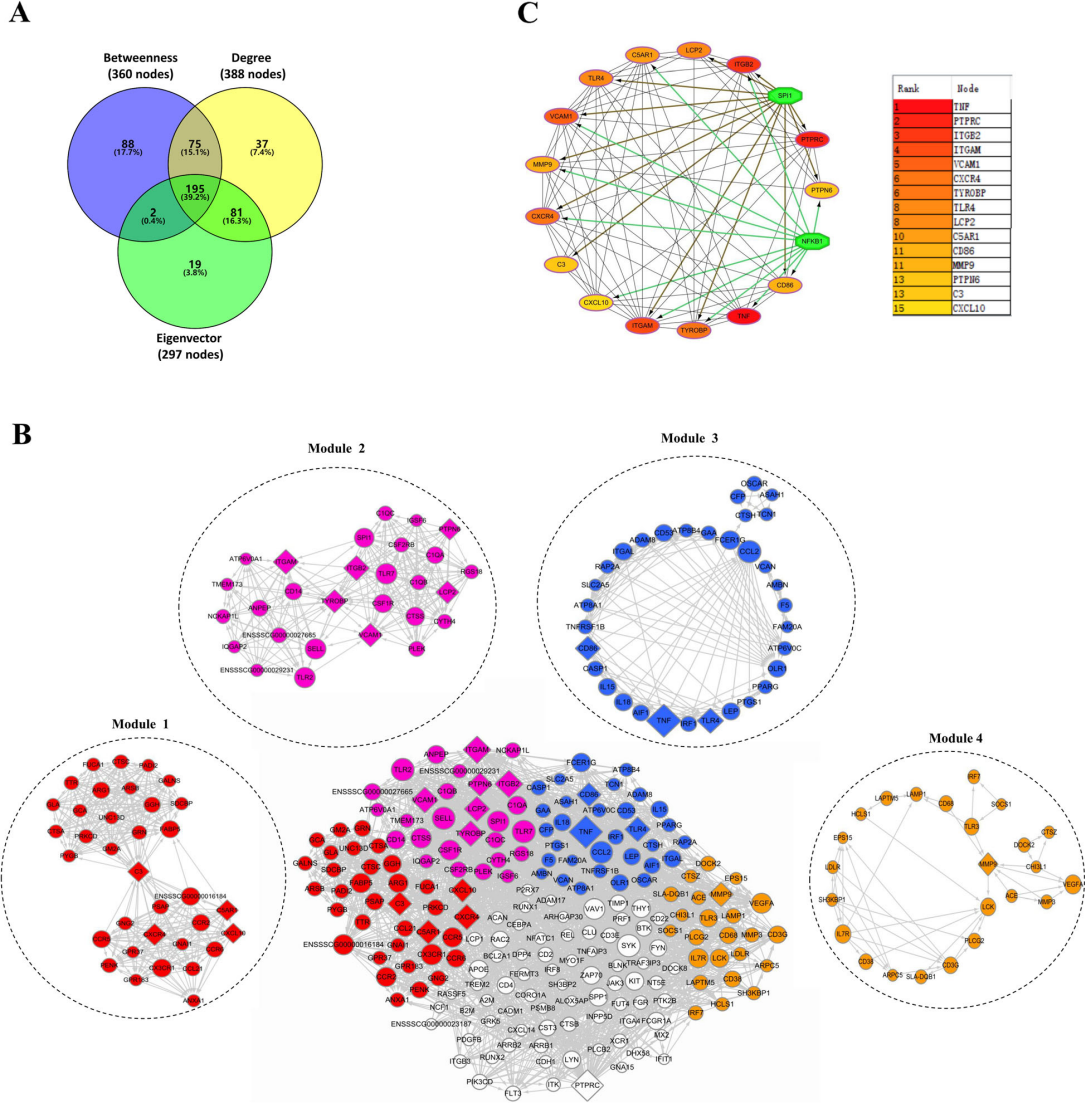
Major PPI networks of DEGs and module analysis. **a** Centrality analysis of the initial PPI network. Venn diagram showing nodes with highest values (above of the average) for each centrality parameter considered (Degree, Betweenness, and eigenvector). **b** Major PPI networks of DEGs and MCODE analysis. Module 1: score = 18.235, module 2: score = 15.259, module 3: score = 8.188; module 4: score = 4.087. Diamond represents a hub gene. Circles sizes represent a degree. Gray arrows indicate a connection between genes. **c** The major PPI network of top 15 hub genes and transcription factor determination shows SPI1 or NFĸB-1 to be important regulators of the top 15 hub genes. Node color reflects the degree of connectivity. Red color represents the highest degree, green color represents a transcription factor

To obtain the major PPI network from the initial network, we performed the following analysis. First, each node’s topological connectivity was determined based on the degree, betweenness, and eigenvector measurement, as shown in Fig. 4a. Three hundred eighty-eight nodes were identified from the PPI network with a degree value above the average (^−^x = 11.549), and were therefore considered as hub nodes. Second, the betweenness of nodes exceeding the average value (^−^x = 3400.411) were considered as bottlenecks, which are the key connector proteins in the PPI network. In total, 360 bottlenecks nodes were identified. Finally, through further analysis of eigenvector parameters, a smaller and more important network was found from the PPI network. There were 297 nodes with an eigenvector value higher than the average (x = 0.013). According to the analysis of centrality parameters, a total of 195 nodes showed high centrality values, which is equivalent to 39.2% of the initial PPI network.

**Fig. 5:**
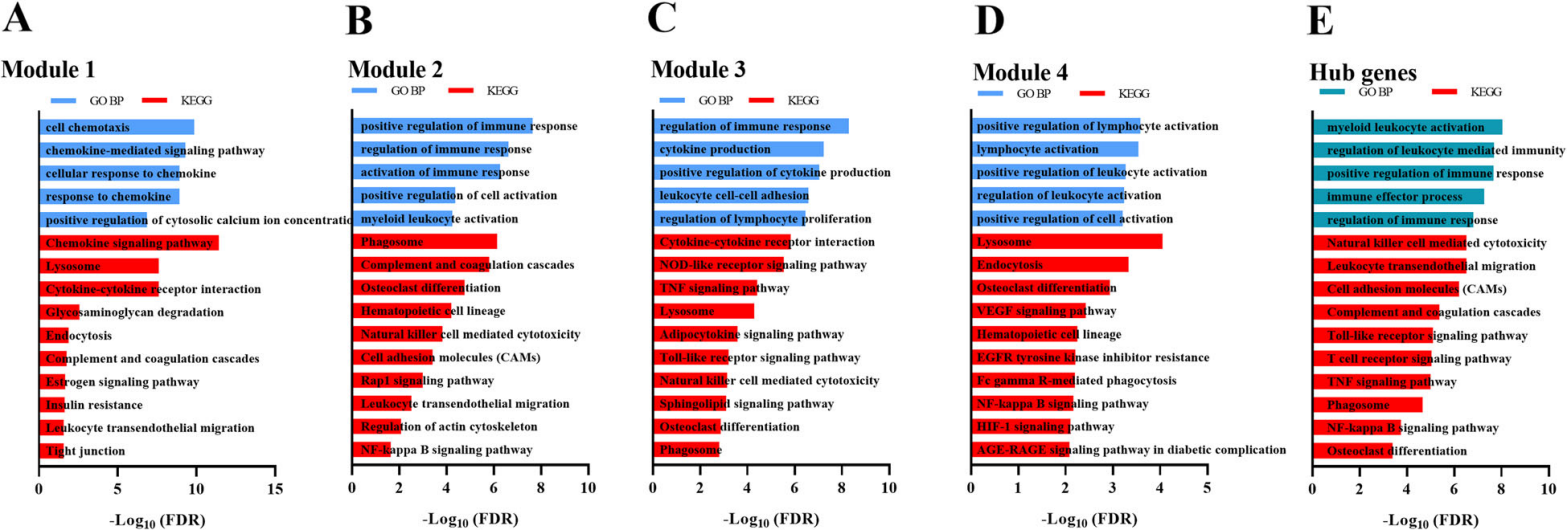
The enriched GO and KEGG pathways of 4 modules and hub genes from major PPI network (**a**-**e**), blue color respected top 5 GO BP, red color respected top 10 KEGG pathways. FDR q value < 0.05

The resulting major PPI network consists of 195 nodes and 2147 edges. To analyze the major PPI network, we identified the top 15 hub genes with higher degree connectivity using the CytoHubba plugin (Fig. 4c), and sequentially ordered them as follows: *TNF* (Degree = 75), *PTPRC* (Degree = 71), *ITGB2* (Degree = 58), *ITGAM* (Degree = 57), *VCAM1* (Degree = 53, *CXCR4* (Degree = 52), *TYROBP* (Degree = 52), *TLR4* (Degree = 50), *LCP2* (Degree = 50), *C5AR1* (Degree = 48), *CD86* (Degree = 47), *MMP9* (Degree = 47), *PTPN6* (Degree = 46), *C3* (Degree = 46), CXCL10 (Degree = 45). The names and functions of these hub genes are shown in Table 3. The iRegulon plugin application in Cytoscape software was used to analyze the promoter binding motifs and to identify the transcription factors associated with each gene. The results show that SPI1 or NFĸB-1 are important regulators of the top 15 hub genes. These 15 hub genes mainly participate in the biological process of myeloid leukocyte activation (Fig. 5e), and are involved in KEGG pathways like natural killer cell mediated cytotoxicity, leukocyte transendothelial migration, cell adhesion molecules (CAMs), complement and coagulation cascades, Toll-like receptor signaling pathway, T cell receptor signaling pathway, TNF signaling pathway, phagosome, NF-kappa B signaling pathway, and osteoclast differentiation (Fig. 5e). Moreover, four significant modules were found via the MCODE plugin (Fig. 4b), with CXCR4, C5AR1, C3 and CXCL10 in module 1, ITGB2, ITGAM, VCAM1, TYROBP, LCP2 and PTPN6 in module 2, TNF, TLR4 and CD86 in module 3, and MMP9 in module 4. Enrichment GO biological processes and pathways of the four modules are displayed in Fig. 5. In these four modules, the most significantly enriched GO biological processes include cell chemotaxis, myeloid leukocyte activation, cytokine production and lymphocyte activation, while the main enriched KEGG pathways include chemokine signaling pathways, insulin resistance, leukocyte transendothelial migration, phagosome, complement and coagulation cascades, osteoclast differentiation, hematopoietic cell lineage, natural killer cell mediated cytotoxicity, cell adhesion molecules (CAMs), cytokine-cytokine receptor interaction, NOD-like receptor signaling pathway, TNF signaling pathway, adipocytokine signaling pathway, Toll-like receptor signaling pathway, lysosome, endocytosis, VEGF signaling pathway, Fc gamma R-mediated phagocytosis, NF-kappa B signaling pathway, and HIF-1 signaling pathway.

**Table 3:** Function summary of the 15 hub genes

No.	Symbol	Description	Function
1	*TNF*	Tumor necrosis factor	This gene encodes a multifunctional proinflammatory cytokine that belongs to the tumor necrosis factor (TNF) superfamily. This cytokine is mainly secreted by macrophages, and is involved in the regulation of a wide spectrum of biological processes including cell proliferation, differentiation, apoptosis, lipid metabolism, and coagulation.
2	*PTPRC*	Protein tyrosine phosphatase receptor type C	The protein encoded by this gene is a member of the protein tyrosine phosphatase (PTP) family. PTPs are known to be signaling molecules that regulate a variety of cellular processes including cell growth, differentiation, mitosis, and oncogenic transformation.
3	*ITGB2*	Integrin subunit beta 2	This gene encodes an integrin beta chain, which combines with different alpha chains to form different integrin heterodimers. The encoded protein plays an important role in immune response, and defects in this gene cause leukocyte adhesion deficiency.
4	*ITGAM*	Integrin subunit alpha M	This gene encodes the integrin alpha M chain. The alpha M beta 2 integrin is important in the adherence of neutrophils and monocytes to stimulated endothelium, and is also important in the phagocytosis of complement coated particles.
5	*VCAM1*	Vascular cell adhesion molecule 1	This gene is a member of the Ig superfamily and encodes a cell surface sialoglycoprotein expressed by cytokine-activated endothelium. This type I membrane protein mediates leukocyte-endothelial cell adhesion and signal transduction, and may play a role in the development of artherosclerosis and rheumatoid arthritis.
6	*CXCR4*	C-X-C motif chemokine receptor 4	This gene encodes a CXC chemokine receptor specific for stromal cell-derived factor-1. The protein has 7 transmembrane regions and is located on the cell surface. It acts with the CD4 protein to support HIV entry into cells and is also highly expressed in breast cancer cells.
7	*TYROBP*	Transmembrane immune signaling adaptor TYROBP	This gene encodes a transmembrane signaling polypeptide which contains an immunoreceptor tyrosine-based activation motif (ITAM) in its cytoplasmic domain. This protein may bind with zeta-chain (TCR) associated protein kinase 70 kDa (ZAP-70) and spleen tyrosine kinase (SYK), and plays a role in signal transduction, bone modeling, brain myelination, and inflammation.
8	*TLR4*	Toll Like receptor 4	The protein encoded by this gene is a member of the Toll-like receptor (TLR) family which plays a fundamental role in pathogen recognition and activation of innate immunity.
9	*LCP2*	Lymphocyte cytosolic protein 2	This gene encodes an adapter protein that acts as a substrate of the T cell antigen receptor (TCR)-activated protein tyrosine kinase pathway. A similar protein plays a role in normal T-cell development and activation. Lacking this gene causes subcutaneous and intraperitoneal fetal hemorrhaging, dysfunctional platelets and impaired viability.
10	*C5AR1*	Complement C5a receptor 1	C5AR1 (Complement C5a Receptor 1) is a protein coding gene. Diseases associated with C5AR1 include hypersensitivity reaction type III disease and mast-cell sarcoma. Its related pathways include signaling by GPCR and innate immune system.
11	*CD86*	CD86 molecule	This gene encodes a type I membrane protein that is a member of the immunoglobulin superfamily. The binding of this protein with CD28 antigen is a costimulatory signal for activating T-cells. Binding of this protein with cytotoxic T-lymphocyte-associated protein 4 negatively regulates T-cell activation and reduces immune response.
12	*MMP9*	Matrix metallopeptidase 9	Proteins of the matrix metalloproteinase (MMP) family are involved in the breakdown of extracellular matrix in normal physiological processes, such as embryonic development, reproduction and tissue remodeling, as well as in disease processes, such as arthritis and metastasis.
13	*PTPN6*	Protein tyrosine phosphatase non-receptor type 6	The protein encoded by this gene is a member of the protein tyrosine phosphatase (PTP) family. PTPs are known to be signaling molecules that regulate a variety of cellular processes including cell growth, differentiation, mitotic cycle, and oncogenic transformation. This PTP is expressed primarily in hematopoietic cells, and functions as an important regulator of multiple signaling pathways in hematopoietic cells.
14	*C3*	Complement C3	Complement C3 plays a central role in the activation of complement system. The C3a peptide, also known as the C3a anaphylatoxin, modulates inflammation and possesses antimicrobial activities.
15	*CXCL10*	C-X-C motif chemokine ligand 10	This antimicrobial gene encodes a chemokine of the CXC subfamily and a ligand for the receptor CXCR3. Binding of this protein to CXCR3 results in pleiotropic effects, including stimulation of monocytes, natural killer and T-cell migration, and modulation of adhesion molecule expression.

### Hub genes validation

**Fig. 6:**
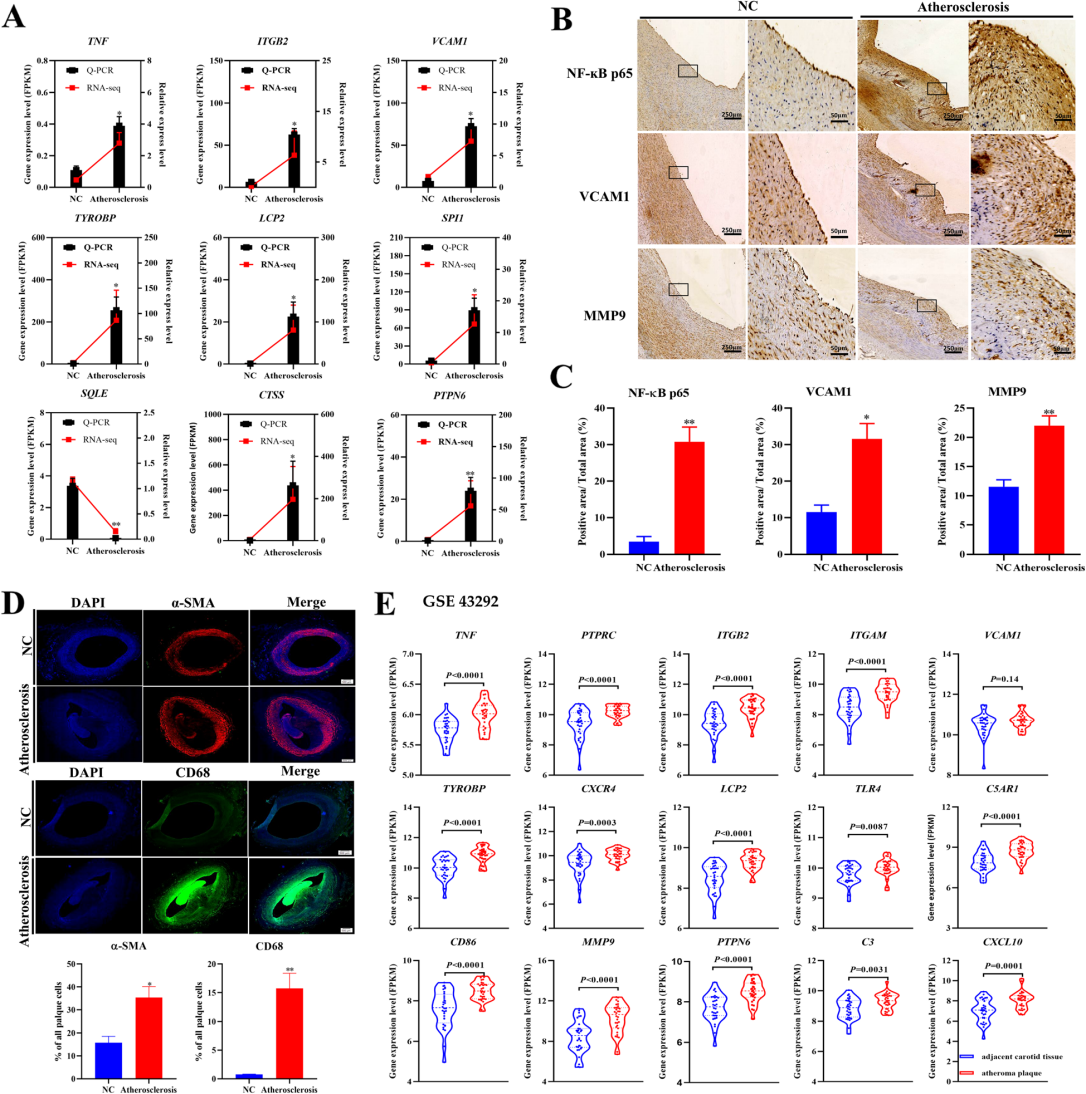
Validation of hub genes. Validation of TNF, *ITGB2*, *VCAM1, TYROBP*, *LCP2*, *SPI1, SQLE, CTSS* and *PTPN6* gene expression levels using RT-PCR. **b** Immunohistochemical staining for NF-ĸB p65, VCAM1, and MMP9 in the abdominal aorta, left, scale bar = 250 μm; and right, scale bar = 50 μm. **c** NF-ĸB p65, VCAM1, and MMP9 protein levels were quantified in atherosclerosis and NC samples. Data are presented as mean ± SEM. **, *P* < 0.01 versus the NC group. **d** α-SMA and CD68 immunofluorescence staining and quantitative analysis results in the abdominal aorta. Scale bar = 500 μm. **e** mRNA expression levels of the top 15 hub genes in human adjacent carotid tissues and atheroma plaque (GSE 43292) data set

To verify the results of RNA-seq data, we selected 9 genes (8 up-regulated and 1 down-regulated) for real-time PCR analysis. We found that the mRNA levels of *TNF*, *ITGB2*, *VCAM1*, *TYROBP, LCP2*, *SPI1*, *CTSS* and *PTPN6* in the atherosclerosis group were significantly increased (*P* < 0.05, *P* < 0.01) compared to the NC group, while the mRNA level of *SQLE* was significantly decreased (*P* < 0.01, Fig. 6a). The RT-PCR results are completely consistent with our RNA-seq results. Immunohistochemical analysis showed positive staining and significantly increased expression of NF-ĸB, VCAM1 and MMP9 proteins in the atherosclerosis group compared to the NC group (*P* < 0.05, *P* < 0.01, Fig. 6b and c). Immunofluorescence staining also showed that the expression of vascular SMCs and CD68 were significantly increased in the atherosclerosis group (*P* < 0.05, *P* < 0.01; Fig. 6d). Furthermore, to confirm whether these 15 hub genes have a similar expression pattern in human atherosclerotic diseases, we used the human carotid plaques (GSE43292) dataset for validation. As shown in Fig. 6e, the mRNA expression levels of the top 15 hub genes are increased in the human atheroma plaque compared to those in the adjacent carotid tissues.

## Discussion

Poor diet quality is a key factor leading to hypercholesterolemia and atherosclerosis. Current guidelines for reducing the risk of cardiovascular diseases also focused on dietary habits. Therefore, animal models that mimic the pathogenesis of human atherosclerosis have become increasingly important in preclinical studies for assessing dietary and drug interventions, as well as for elucidating their underlying mechanism. Our results showed that Tibetan minipigs developed dyslipidemia and immune inflammation accompanied by early-stage atherosclerosis after the consumption of a HFC diet relative to a regular diet. The main pathological changes are characterized by lipid deposition, hyperplasia of foam cells, smooth muscle cells and fiber-matrix, as well as calcification, which is similar to the pathogenesis of human atherosclerosis lesions. This provides a reliable model for studying the relationship between diet and atherosclerosis. Therefore, the pathogenesis of the Tibetan minipig atherosclerosis model deserves further study.

Atherosclerosis is a slowly progressive disease accompanied by inflammation, and inflammatory and immune responses are important links in its formation. High-throughput sequencing can provide a new way for studying the pathogenesis of atherosclerosis. In this study, 1716 DEGs were identified that include 1468 up-regulated DEGs and 248 down-regulated DEGs. GO biological process enrichment, KEGG pathway, PPI network, and module analysis were helpful to find key genes and pathways in Tibetan minipigs with atherosclerosis. GO biological process enrichment analysis showed that up-regulated DEGs are mainly related to immune-inflammatory responses, cell proliferation, differentiation and chemotaxis, response to lipids, reactive oxygen species metabolic process and cell death, which confirmed that cell proliferation and differentiation, cell adhesion, immune inflammation, and lipid metabolism are well associated with atherosclerosis [25]. Meanwhile, our analysis showed that the down-regulated DEGs are involved in muscle contraction, which is in agreement with the results in human carotid atherosclerotic plaque from Liu et al. [26]. These results supported that lipid metabolism, oxidative stress and immune-inflammatory responses drive the development of atherosclerosis, which is consistent with the enrichment analysis results of hallmark gene sets in GSEA.

After the HFC diet, the LDL-C level of Tibetan minipigs was significantly increased with increased leukocyte recruitment. It can be seen that the increase of lipids (especially LDL) and leukocytes are key causes of the chronic inflammation of atherosclerosis. There was increasing evidence that LDL stimulates innate and adaptive immunity in atherosclerosis [27, 28]. Similar results were also confirmed in KEGG pathways enrichment analysis, where phagosomes, lysosomes, cytokine-cytokine receptor interactions, chemokine signaling pathway, complement and coagulation cascades, and antigen processing and presentation were shown to participate in the activation of the adaptive immune response by innate immune cells [29]. At the same time, T and B cell receptor signaling pathways associated with the adaptive immune responses were also enriched and involved in atherosclerosis and arterial thrombosis in humans [28]. Natural killer cell-mediated cytotoxicity was also known to play a role in both innate and adaptive immune-mediated responses [29]. In addition, the Toll-like receptor signaling pathway, NF-kappa B signaling pathway and JAK-STAT signaling pathway have been confirmed as atherosclerosis-related inflammation signaling pathways [30]. Therefore, we believe that a HFC diet induces hypercholesterolemia in Tibetan minipigs and activates innate and adaptive immunity to drive the development of atherosclerosis. Interestingly, it was also found that insulin resistance pathways are significantly enriched in blood vessels, which is consistent with our early study showing that the HFC diet induces insulin resistance and promotes vascular insulin resistance in Tibetan minipigs [9].

To further understand the molecular mechanism of HFC diet-induced atherosclerosis in Tibetan minipigs, a representative PPI network of atherosclerosis was obtained. We first constructed an initial PPI network of 1716 DEGs. Based on the degree, betweenness and eigenvector analysis of the initial PPI network topology, 195 DEGs were selected to create a high centrality major PPI network, indicating the decisive roles of these proteins in atherosclerosis induced by HFC-diet in Tibetan minipigs. Four key modules were obtained by MCODE cluster analysis, in which we saw that the genes in these modules are mainly involved in the biological process of cell chemotaxis, myeloid leukocyte activation, cytokine production, and lymphocyte activation. Moreover, a total of 15 hub genes were extracted from the major PPI network through degree centrality analysis, including TNF, PTPRC, ITGB2, ITGAM, VCAM1, CXCR4, TYROBP, TLR4, LCP2, C5AR1, CD86, MMP9, PTPN6, C3, and CXCL10. GO biological process enrichment analysis revealed that these hub genes are mainly involved in myeloid leukocyte activation. Previous studies have shown that a variety of myeloid cells (monocytes, macrophages, dendritic cells, and neutrophils) in the aorta are involved in regulating the progression of inflammation and atherosclerosis [31], and suggested that the activation of myeloid leukocytes can promote vascular endothelial injury and inflammatory response, and accelerate the progression of atherosclerosis. For example, monocytes can differentiate into macrophages and swallow lipids, form foam cells, and secrete a variety of inflammatory factors such as TNF-α, interleukin-1 (IL-1) and IL-6, leading to cell adhesion, inflammatory cell infiltration, matrix degradation, and ultimately plaque rupture [32]. Among the hub genes, TNF, PTPRC (also known as CD45), VCAM1, CD86 and MMP9 have been confirmed as inflammatory biomarkers of atherosclerosis [33, 34]. Chemokines are known to play a crucial role in atherosclerosis, especially in the recruitment of monocytes. CXCR4 is widely expressed not only on the surface of hematopoietic stem cells but also in atherosclerotic lesions. Animal experiments have shown that CXCR4 expression was significantly up-regulated in the thoracic aorta vessels of high-fat diet-fed ApoE^−/−^ mice [35]. Heller EA et al. [36] showed that lymphocytes indeed promote atherosclerosis, and identified CXCL10, a T cell chemokine which enhances this process. Complement and coagulation cascades are the main defense systems that mediate inflammation and thrombosis. Hypercholesterolemia can activate complement to produce C3 invertase through the classical pathway, lectin pathway or alternative pathway. At the same time, the downstream of C5 invertase was formed to lyse C5 and form C5a [37]. Patzelt J et al. [38] found that the expression of C3aR and C5aR in patients with coronary heart diseases was positively correlated with platelet activation. Recently, NK cells, especially NKT cells, have been identified as important participants in immune metabolism because of their unique responses to lipid antigens and their mixed nature of innate and adaptive immune systems [39]. ITGB2 (integrin subunit beta 2, CD18) is an adhesion molecule of the integrin family and a major marker of NK cell maturity, and is involved in adhesion between inflammatory cells and endothelial cells, inflammatory cells chemotaxis, and other processes that are active in the early stages of atherosclerosis [40]. ITGAM encodes the integrin αM chain that is involved in CD40L-mediated inflammation during atherosclerosis [41]. TYROBP (also known as DNAX activator protein 12, DAP12) is a kind of transmembrane receptors that is widely present in natural killer cells, neutrophils and monocytes/macrophages. Wang et al. [42] found that DAP12 was highly exhibited in plaques of APOE mice and promoted the formation of atherosclerosis through the TREM-1/DAP12 pathway. Type 6 protein tyrosine phosphatase non-receptor (PTPN6, also named as SHP-1) is a member of the protein tyrosine phosphatase (PTP) family located in the cytoplasm, which regulates cell growth, differentiation, mitotic cycle and oncogenic transformation [43]. PTPN6 plays a vital role in negatively regulating insulin action and liver clearance, thereby regulating systemic glucose homeostasis [44]. Nai et al. [45] found that PTPN6 was significantly up-regulated in atherosclerosis. LCP2, also known as SLP-76, is an adaptor protein specific to blood-derived cells, which is mainly present in the hematopoietic system and platelets, NK cells, neutrophils, mast cells, immature B cells and macrophages, and participates in T cell receptor-related signaling pathways and coagulation function [46]. It can be seen that the activation of these hub genes is involved in promoting the adhesion between inflammatory cells and endothelial cells, and in accelerating the formation of atherosclerotic plaques.

In addition, TLR4, a member of pattern recognition receptors family, plays a key role in innate immunity and has recently received widespread attention. In monocytes, TLR4 and TNF-α can activate the classic NF-κB/AP-1 signaling pathway and promote the release of inflammatory cytokines [47]. This is consistent with our findings about the enrichment of TNF, TLR, and NF-kappa B pathways. We found that 11 hub genes are regulated by the NF-ĸB-1 transcription factor, which was validated by the high expression of NF-ĸB in atherosclerotic vessels in Tibetan minipigs from immunohistochemical staining. On the other hand, 12 hub genes are controlled by the SPI1 transcription factor (spleen focus forming virus proviral integration oncogene), which is a member of the ETS transcription factor family. Previous studies have shown that the mRNA level of SPI1 is up-regulated during myeloid cell differentiation and is maintained at high levels in human B cells, mast cells, monocytes, peripheral blood neutrophils and other granulocytes [48]. This is consistent with our observation of the significant increase of SPI1 expression in Tibetan minipigs induced by the HFC diet, further suggesting that the activation of myeloid cells plays a very important role in the formation of atherosclerosis.

To ensure the accuracy of candidate DEGs, we selected 9 genes for RT-PCR validation. The results confirmed the consistency between the gene expression and sequencing results. Furthermore, we used the human carotid atherosclerosis dataset (GSE 43292) to validate the expression pattern of 15 hub genes in human carotid plaques. We found that the expression patterns of these 15 hub genes in human carotid artery plaques agree with those in atherosclerotic vessels of Tibetan minipigs induced by the HFC diet, suggesting that the HFC diet-induced Tibetan minipigs atherosclerosis model is suitable for the study of atherosclerosis-related diseases in humans.

## Conclusion

Our data showed that the HFC diet-induced Tibetan minipig atherosclerosis model exhibits significant dyslipidemia, oxidative stress, immune-inflammatory responses, and increased LDL and leukocyte recruitment. Our RNA-seq experiment revealed 1716 DEGs in the atherosclerosis/NC group, including 1468 up-regulated genes and 248 down-regulated genes. Functional enrichment analysis of DEGs showed that the HFC diet induced changes are related to vascular immune-inflammatory responses, lipid metabolism and muscle contraction, indicating that HFC diet-induced hypercholesterolemia activates innate and adaptive immunity to drive atherosclerosis development. Four modules, associated with cell chemotaxis, myeloid leukocyte activation, cytokine production, and lymphocyte activation, were identified from the major PPI network. Fifteen hub genes were discovered, including TNF, PTPRC, ITGB2, ITGAM, VCAM1, CXCR4, TYROBP, TLR4, LCP2, C5AR1, CD86, MMP9, PTPN6, C3, and CXCL10, as well as two transcription factors, i.e. NF-ĸB1 and SPI1. This bioinformatics study has identified the hub genes and key pathways for the development of atherosclerosis in Tibetan minipigs induced by a HFC diet.

## Data Availability

The datasets used and analysed during the current study available from the corresponding author on reasonable request.

## Abbreviations

AI: Atherogenic index
BP: Biological process
C5AR1: Complement C5a receptor 1
CRP: C reactive protein
CXCL10: C-X-C motif chemokine ligand 10
CXCR4: C-X-C motif chemokine receptor 4
DEGs: Differentially expressed genes
FDR: False discovery rate
GO: Gene ontology
GSEA: Gene Set Enrichment Analysis
H&E: Hematoxylin and Eosin
HDL-C: High density lipoprotein cholesterol
HFC: High fat/cholesterol
IMT: Intima-media thickness
ITGAM: Integrin subunit alpha M
ITGB2: Integrin Beta 2
KEGG: Kyoto Encyclopedia of Genes and Genomes
KOBAS: KEGG Orthology Based Annotation System
LCP2: Lymphocyte cytosolic protein 2
LDL-C: Low density lipoprotein cholesterol
MCODE: Molecular complex detection
MMP9: Matrix metallopeptidase 9
MSigDB: Molecular Signature Database
NC: Normal control
NES: Normalized enrichment score
PPI: Protein-protein interactions
PTPN6: Protein tyrosine phosphatase non-receptor type 6
PTPRC: Recombinant Protein Tyrosine Phosphatase Receptor Type C
SMCs: Smooth muscle cells
SPI1: Spleen focus forming virus proviral integration oncogene
STRING: Search Tool for the Retrieval of Interacting Genes
TC: Total cholesterol
TF: Transcription factors
TG: Triglycerides
TLR4: Toll like receptor 4
TNF: Tumor necrosis factor
TYROBP: TYRO protein tyrosine kinase binding protein
VCAM1: Vascular cell adhesion molecule 1
WBC: White blood cell
